# Intravenous administration of BCG protects mice against lethal SARS-CoV-2 challenge

**DOI:** 10.1084/jem.20211862

**Published:** 2021-12-10

**Authors:** Kerry L. Hilligan, Sivaranjani Namasivayam, Chad S. Clancy, Danielle O’Mard, Sandra D. Oland, Shelly J. Robertson, Paul J. Baker, Ehydel Castro, Nicole L. Garza, Bernard A.P. Lafont, Reed Johnson, Franca Ronchese, Katrin D. Mayer-Barber, Sonja M. Best, Alan Sher

**Affiliations:** 1 Immunobiology Section, Laboratory of Parasitic Diseases, National Institute of Allergy and Infectious Diseases, National Institutes of Health, Bethesda, MD; 2 Immune Cell Biology Programme, Malaghan Institute of Medical Research, Wellington, New Zealand; 3 Rocky Mountain Veterinary Branch, National Institute of Allergy and Infectious Diseases, National Institutes of Health, Hamilton, MT; 4 Innate Immunity and Pathogenesis Section, Laboratory of Virology, Rocky Mountain Laboratories, National Institute of Allergy and Infectious Diseases, National Institutes of Health, Hamilton, MT; 5 Inflammation and Innate Immunity Unit, Laboratory of Clinical Immunology and Microbiology, National Institute of Allergy and Infectious Diseases, National Institutes of Health, Bethesda, MD; 6 SARS-CoV-2 Virology Core, Laboratory of Viral Diseases, National Institute of Allergy and Infectious Diseases, National Institutes of Health, Bethesda, MD

## Abstract

In addition to providing partial protection against pediatric tuberculosis, vaccination with bacille Calmette-Guérin (BCG) has been reported to confer nonspecific resistance to unrelated pulmonary pathogens, a phenomenon attributed to the induction of long-lasting alterations within the myeloid cell compartment. Here, we demonstrate that intravenous, but not subcutaneous, inoculation of BCG protects human-ACE2 transgenic mice against lethal challenge with SARS-CoV-2 (SCV2) and results in reduced viral loads in non-transgenic animals infected with an α variant. The observed increase in host resistance was associated with reductions in SCV2-induced tissue pathology, inflammatory cell recruitment, and cytokine production that multivariate analysis revealed as only partially related to diminished viral load. We propose that this protection stems from BCG-induced alterations in the composition and function of the pulmonary cellular compartment that impact the innate response to the virus and ensuing immunopathology. While intravenous BCG vaccination is not a clinically acceptable practice, our findings provide an experimental model for identifying mechanisms by which nonspecific stimulation of the pulmonary immune response promotes host resistance to SCV2 lethality.

## Teaser

BCG when administered by the intravenous but not subcutaneous route provides protection against lethal SARS-CoV-2 infection in mice, reducing immunopathology in addition to viral load and thus providing an experimental model for delineating innate mechanisms of resistance against COVID-19.

## Introduction

COVID-19 is a pneumonic disease caused by the newly emerged coronavirus severe acute respiratory syndrome coronavirus 2 (SCV2). A wide spectrum of disease severity is observed in humans, with some patients remaining entirely asymptomatic and others progressing to severe respiratory disease, multiple organ failure, and death (Hu et al., 2021). The symptoms and pathology associated with severe forms of COVID-19 are thought to be driven by an overzealous and sustained innate immune response, with disease severity positively correlated with high levels of proinflammatory cytokines, NLRP3 inflammasome assembly, and myeloid cell activation (Del Valle et al., 2020; Delorey et al., 2021* Preprint*; Melms et al., 2021; Thwaites et al., 2021; Vora et al., 2021).

IFN-Is are important mediators of antiviral responses, and suboptimal or delayed IFN-I responses are associated with more severe forms of COVID-19 (Hadjadj et al., 2020; O’Brien et al., 2020; Zhang et al., 2020). However, prolonged production of IFN-I, IFN-λ, and other innate cytokines, such as IL-6 and TNF-α, can contribute to pulmonary pathology through induction of cell death pathways, recruitment of proinflammatory cells, and disruption of epithelial repair, as well as to vascular damage through pleiotropic effects on endothelial, vascular smooth muscle cells, and infiltrating leukocytes (Channappanavar et al., 2016; Israelow et al., 2020; Karki et al., 2021; Major et al., 2020; Ragab et al., 2020; Thacker et al., 2012). Induction of early innate cytokines followed by a tempering of this response may therefore be important determinants of disease outcome. Indeed, prior proinflammatory events can be protective against subsequent unrelated infections through preexisting IFN priming or the recruitment of antimicrobial monocyte-derived cells into the airways (Aegerter et al., 2020; Cheemarla et al., 2021). CCR2-dependent cells, such as monocytes and type 2 dendritic cells (DC2s), support early viral control in SCV2 infection models, with CCR2-KO mice displaying higher viral titers in the lungs 4 d postinfection (dpi; Vanderheiden et al., 2021).

Bacille Calmette-Guérin (BCG) is a live attenuated vaccine that is widely used to prevent disseminated tuberculosis in infants and children (Zwerling et al., 2011). In addition to partially protecting against pediatric tuberculosis, BCG vaccination is associated with beneficial, nonspecific effects, including lower all-cause mortality in infants (Biering-Sørensen et al., 2017), reduced viremia after a yellow fever vaccine challenge in adults (Arts et al., 2018), and reduced risk of respiratory infections in the elderly (Giamarellos-Bourboulis et al., 2020). BCG has also proved successful in stimulating antitumor immune responses and is an effective treatment for some forms of bladder cancer (Pettenati and Ingersoll, 2018). These nonspecific effects of BCG have been attributed to epigenetic and metabolic reprogramming of the innate immune system (Arts et al., 2016; Kleinnijenhuis et al., 2012), as well as to the redirection of hematopoiesis toward the rapid generation of protective myeloid subsets (Brook et al., 2020; Cirovic et al., 2020; Kaufmann et al., 2018). In experimental models, this is particularly evident when BCG is administered by the i.v. as opposed to the more conventional s.c. route (Kaufmann et al., 2018). In this regard, the i.v. route of BCG inoculation has also been shown to provide superior protection against *Mycobacterium tuberculosis* infection in nonhuman primates (Darrah et al., 2020; Sharpe et al., 2016).

Because of its well-described association with nonspecific innate protection, BCG administration has been suggested as a possible prophylactic measure for the prevention of SCV2 infection and disease (O’Neill and Netea, 2020). Indeed, a number of ecological studies have suggested an association of prior BCG vaccination with lower incidence of COVID-19 disease (Escobar et al., 2020; Rivas et al., 2021). This concept, which remains controversial, is currently being formally tested in a number of clinical trials (Junqueira-Kipnis et al., 2020; Pépin et al., 2021; Tsilika et al., 2021* Preprint*). In addition, recent preclinical work has demonstrated the use of BCG as an adjuvant to boost SCV2-specific vaccine-induced protection (Counoupas et al., 2021).

Here, we have systematically evaluated the effects of prior BCG inoculation on SCV2 pathogenesis in two murine experimental models. The first model employs K18-hACE2 mice that are highly susceptible to lethal infection due to their expression of a transgene for the human ACE2 receptor under control of the keratin-18 promoter (McCray et al., 2007). The second model involves challenge with an α B.1.1.7 variant that is able to productively infect WT, non-transgenic animals. We demonstrate that i.v., but not s.c., delivery of BCG confers a high level of protection against SCV2 in both models. The observed BCG-induced resistance is associated with reduced viral titers and proinflammatory cytokine production, as well as decreased pulmonary pathology. We propose that in this experimental setting, i.v. administration of BCG limits the course of SCV2 infection through its targeting of innate immune pathways and, thus, may be a useful platform for identifying early immunological events affecting the outcome of this disease.

## Results and discussion

### Intravenous BCG protects K18-hACE2 animals from lethal SCV2 challenge

**Figure 1.**
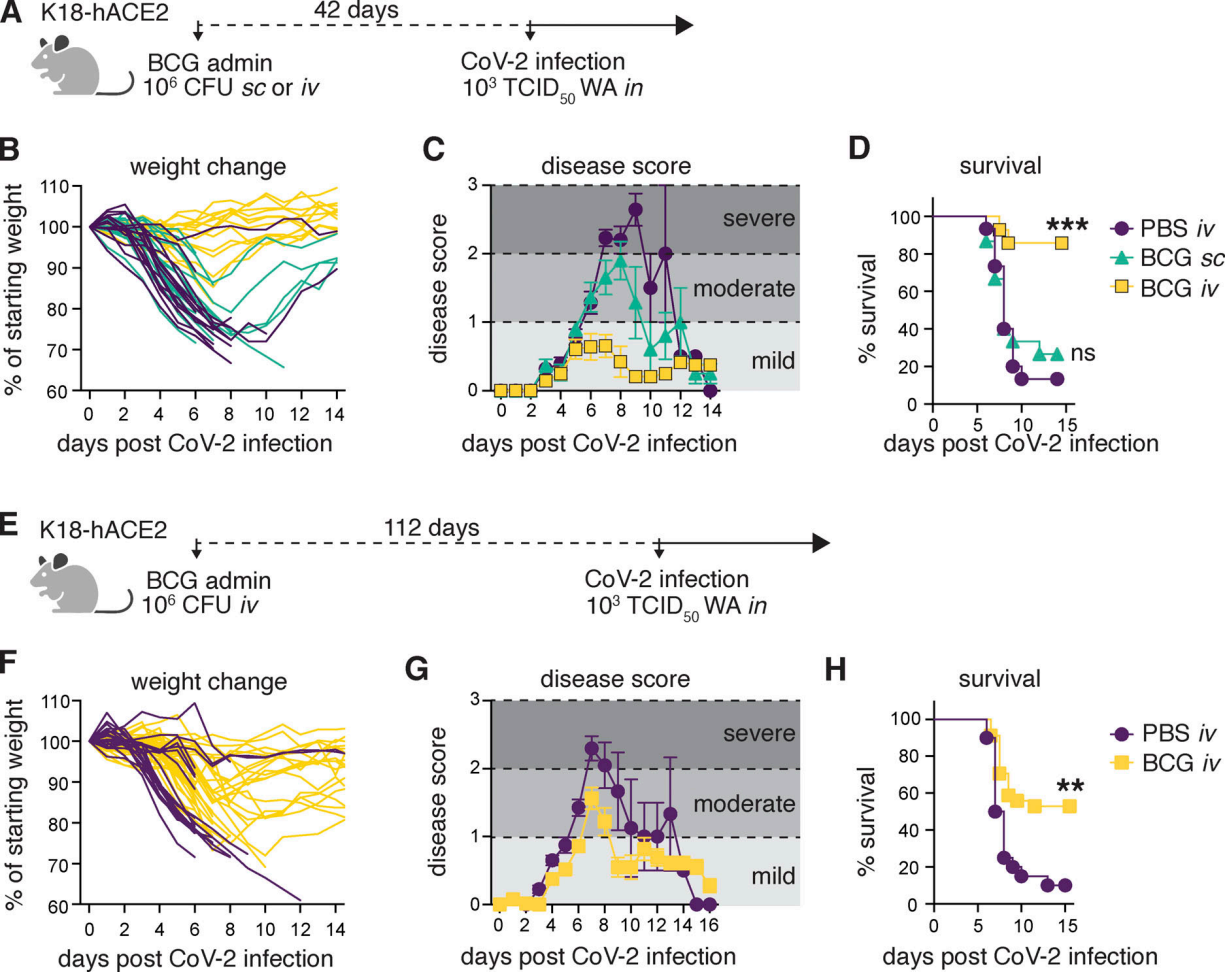
**BCG i.v. administration protects K18-hACE2 mice from lethal SCV2 challenge. (A–H)** K18-hACE2 mice were inoculated with 10^6^ CFUs BCG Pasteur by s.c. or i.v. injection. Control animals received the same volume of PBS i.v. At 42 (A–D) or 112 d (E–H) after BCG administration, mice were infected with 10^3^ TCID_50_ SCV2 WA by i.n. instillation. **(A and E)** Schematic of experimental design. **(B and F)** Weight change following SCV2 infection shown as percentage of starting weight. **(C and G)** Animals were scored on a scale of 0–3 by a blinded observer, with 0 referring to no observable signs of disease and 3 referring to a moribund state requiring euthanasia. Scoring criteria are outlined in the Materials and methods. **(D and H)** Kaplan–Meier curve of animal survival following SCV2 challenge. Statistical significance was assessed by Mantel-Cox test. ns, P > 0.05; **, P < 0.01; ***, P < 0.001. Mean ± SEM is shown. Data are pooled from two to three independent experiments each with 4–15 mice per group.

Nonspecific BCG-induced protection is reported to act by promoting the generation of primed innate immune subsets, which is achieved most efficiently if BCG is administered i.v. as opposed to the more conventional s.c. route (Kaufmann et al., 2018). We therefore hypothesized that if BCG can promote host resistance to SCV2, i.v. administration would be more effective than BCG delivered by alternative routes. To test this, K18-hACE2 transgenic mice were inoculated with BCG either s.c. or i.v. and then challenged with a lethal dose of the USA-WA1/2020 (WA) SCV2 isolate 42 d later (Fig. 1 A). Control animals that received PBS i.v. and mice given BCG s.c. underwent profound weight loss after SCV2 challenge and exhibited clinical signs of severe disease (ruffled fur, hunched posture, lethargy), progressing to a moribund state. In contrast, animals inoculated with BCG i.v. 42 d before SCV2 challenge maintained their body weight and displayed minimal signs of disease as assessed by a blinded observer (Fig. 1, B and C). Accordingly, most animals in the PBS i.v. and BCG s.c. groups succumbed to infection by 12 dpi, whereas BCG i.v. mice were largely protected, with 85% of animals surviving long term (Fig. 1 D). The few surviving control animals recovered their body weight by 15 dpi and, as expected, were fully protected upon SCV2 rechallenge (data not shown).

To assess the longevity of BCG i.v.–induced protection, we challenged animals with virus 112 d after BCG inoculation (Fig. 1 E). BCG i.v. significantly improved survival (50%) compared with PBS-treated controls (10%); however, the level of protection against weight loss, disease, and death was less pronounced than when mice were challenged at 42 d (Fig. 1, F–H), indicating that BCG i.v.–induced protection wanes over time. Together, these data demonstrate that BCG can trigger potent host resistance against SCV2 lethality and that the i.v. route of BCG delivery is critical for determining the protection observed.

### Intravenous BCG reduces SCV2 viral loads in the lungs of K18-hACE2 and WT (non-transgenic) mice

**Figure 2.**
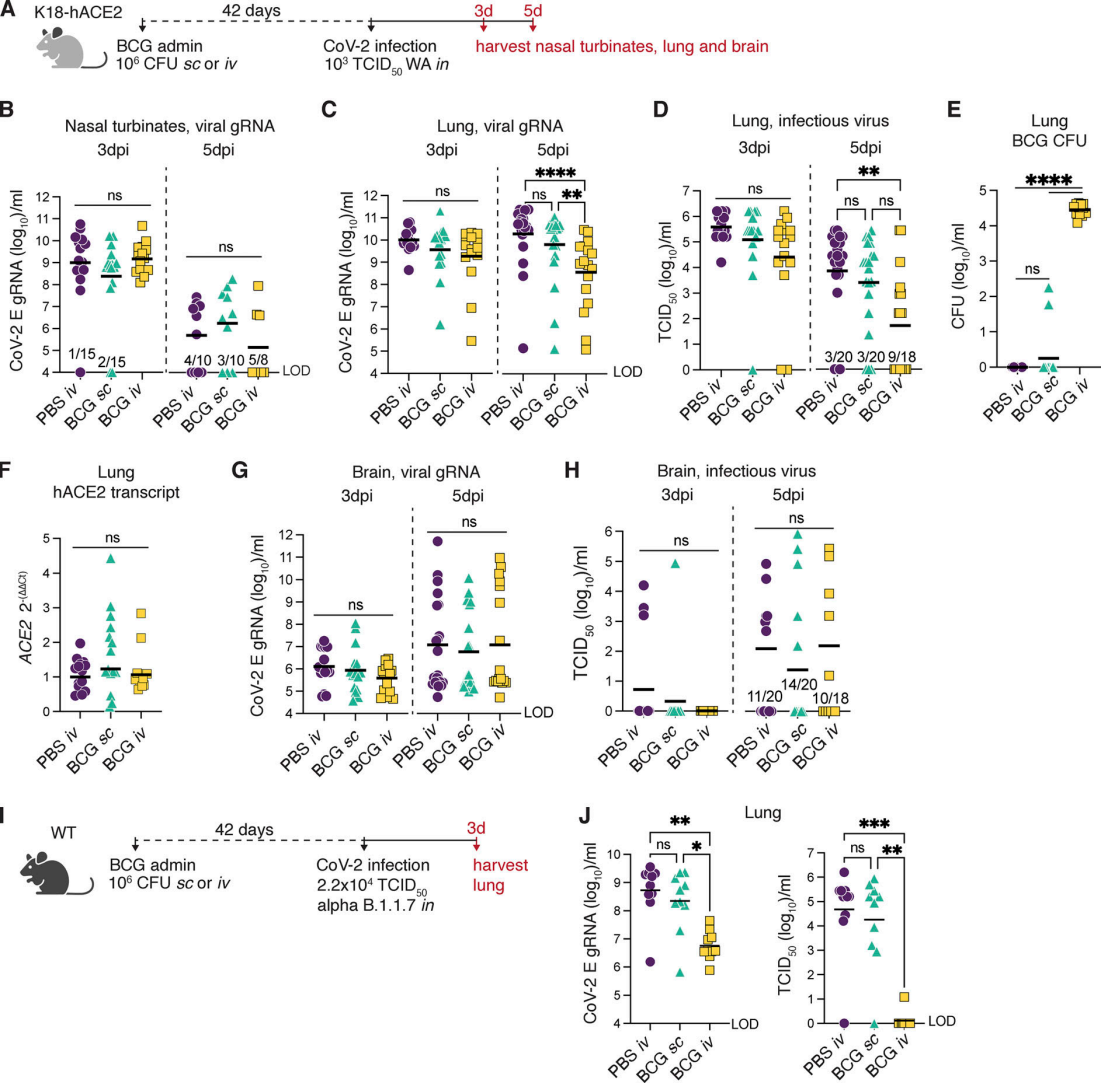
**BCG i.v. administration accelerates pulmonary viral clearance in K18-hACE2 and WT mice. (A–H)** K18-hACE2 mice were inoculated with 10^6^ CFUs BCG Pasteur by s.c. or i.v. injection. Control animals received the same volume of PBS i.v. At 42 d after BCG administration, mice were infected with 10^3^ TCID_50_ SCV2 (WA1/2020) by i.n. instillation. **(B)** Nasal turbinates were collected 3 or 5 d after challenge and assessed for viral load by qPCR. **(C–F)** Lungs were collected 3 or 5 d after viral challenge and assessed for viral load by qPCR and TCID_50_ assay (C and D), mycobacterial load by counting CFUs (E), and expression of human ACE2 by RT-qPCR (F).** (G and H)** Viral loads were quantified in brain samples at 3 and 5 dpi by qPCR and TCID_50_ assay. **(I and J)** C57BL/6 (WT) mice were inoculated with 10^6^ CFUs BCG Pasteur by s.c. or i.v. injection. Control animals received the same volume of PBS i.v. At 42 d after BCG inoculation, mice were infected with 2.2 × 10^4^ TCID_50_ SCV2 (α B.1.1.7) by i.n. instillation. Lungs were collected 3 d after viral challenge, and homogenates were assessed for viral load by qPCR and TCID_50_ assay (J). Statistical significance was assessed by Kruskal-Wallis test with Dunn’s post-test. ns, P > 0.05; *, P < 0.05; **, P < 0.01; ***, P < 0.001; ****, P < 0.0001. Data are pooled from two to four independent experiments each with 4–10 mice per group. Graphs show geometric mean and individual values for each animal. LOD, limit of detection.

To determine whether BCG-induced protection against SCV2 correlates with reduced viral loads within tissues, we harvested nasal turbinates, lung, and brain from BCG-immunized mice at 3 or 5 dpi with SCV2 (Fig. 2 A). Viral loads were measured by the level of viral genomic RNA (gRNA) specific for the E gene by RT quantitative PCR (qPCR) and corroborated by tissue culture infectious dose 50 (TCID_50_) assay for lung and brain samples. gRNA was detectable in the nasal turbinates at 3 dpi but in only half of the animals assessed at 5 dpi. Viral levels in the turbinates did not differ between BCG-immunized and PBS-treated controls among the animals with detectable viral RNA (Fig. 2 B). In the lung, gRNA was detectable in all samples tested and was similar across treatment groups at the 3 dpi time point but at 5 dpi was statistically lower (≥2 logs) in animals inoculated with BCG i.v. before SCV2 challenge compared with PBS controls (Fig. 2, C and D). Assessment of the same lung homogenate samples by TCID_50_ assay confirmed our gRNA data: The amount of infectious virus was not significantly different at 3 dpi but was reduced in the BCG i.v. mice compared with the PBS i.v. treatment group at 5 dpi, with 50% of samples obtained from BCG i.v.–treated animals having no measurable infectious virus at this time point (Fig. 2, C and D). In contrast, animals inoculated with BCG s.c. showed a small, but insignificant reduction in viral loads compared with PBS i.v. controls, which were higher than those recovered from mice administered BCG i.v., although this did not reach statistical significance in the case of the TCID_50_ assay (Fig. 2 D). As expected, enumeration of BCG CFUs showed significantly higher mycobacterial burdens in the lungs of animals that received BCG by the i.v. compared with the s.c. route (Fig. 2 E). Expression of the hACE2 transgene was consistent across treatment groups, suggesting that the lower infectivity was not due to BCG-induced alterations in transcription of the transgene or selective loss of ACE2-expressing host cells (Fig. 2 F). These data demonstrate that SCV2 productively infects and replicates in K18-hACE2 animals regardless of BCG immunization status but that the virus is controlled more effectively in mice previously inoculated with BCG by the i.v. route.

K18-hACE2 mice are well documented to support neurotropism of SCV1 and SCV2, which could contribute to mortality in this model (McCray et al., 2007; Yinda et al., 2021). To determine whether the enhanced survival of K18-hACE2 animals conferred by BCG i.v. was associated with reduced viral loads within the central nervous system (CNS), we assessed gRNA and TCID_50_ titer in brain homogenate 3 or 5 dpi with SCV2. High viral titers (>10^3^ TCID_50_/ml) were observed in a few animals from each treatment group at the 5 dpi time point, but there was no difference in viral RNA or infectious virus across the experimental groups (Fig. 2, G and H), arguing against the possibility that BCG i.v. improves host resistance against SCV2 lethality by modulating viral dissemination to the CNS.

Given the artificial expression and tissue distribution of SCV2 permissive receptors in K18-hACE2 transgenic mice and the atypical CNS dissemination observed in this model, we tested the efficacy of BCG i.v. against SCV2 in an alternate non-transgenic mouse model. WT mice do not support infection and replication by the originally identified SCV2 strains (including the WA isolate) due to incompatibility between the murine ACE2 receptor and the viral spike protein (Zhou et al., 2020). However, recently identified SCV2 variants, including the α B.1.1.7 variant, have acquired mutations in the spike protein that allow for more efficient engagement of murine ACE2 for viral entry (Montagutelli et al., 2021 *Preprint*). We confirmed that WT B6 mice could indeed support replication of the B.1.1.7 variant for 3–4 d before the virus cleared (data not shown). Importantly, this viral expansion was dramatically impaired if the animals had received BCG i.v. before challenge (Fig. 2, I and J).

Together, these data demonstrate that BCG i.v. promotes control of SCV2 replication in two mouse models. The high level of gRNA copies present in the lungs of BCG i.v. mice suggests that these animals were productively infected with SCV2 but were more efficient at controlling viral replication and infectivity, as evidenced by lower infectious viral titers recovered from the lungs at 5 dpi. Nevertheless, it was unclear whether the observed level of viral control could account for the striking protection from SCV2-driven mortality conferred by BCG i.v. seen in K18-hACE2 mice. To further examine this issue, we performed a more detailed analysis of the pulmonary immune landscape to identify correlates of protection.

### Intravenous inoculation with BCG reduces SCV2-associated pulmonary pathology, immune cell infiltration, and chemokine production

**Figure 3.**
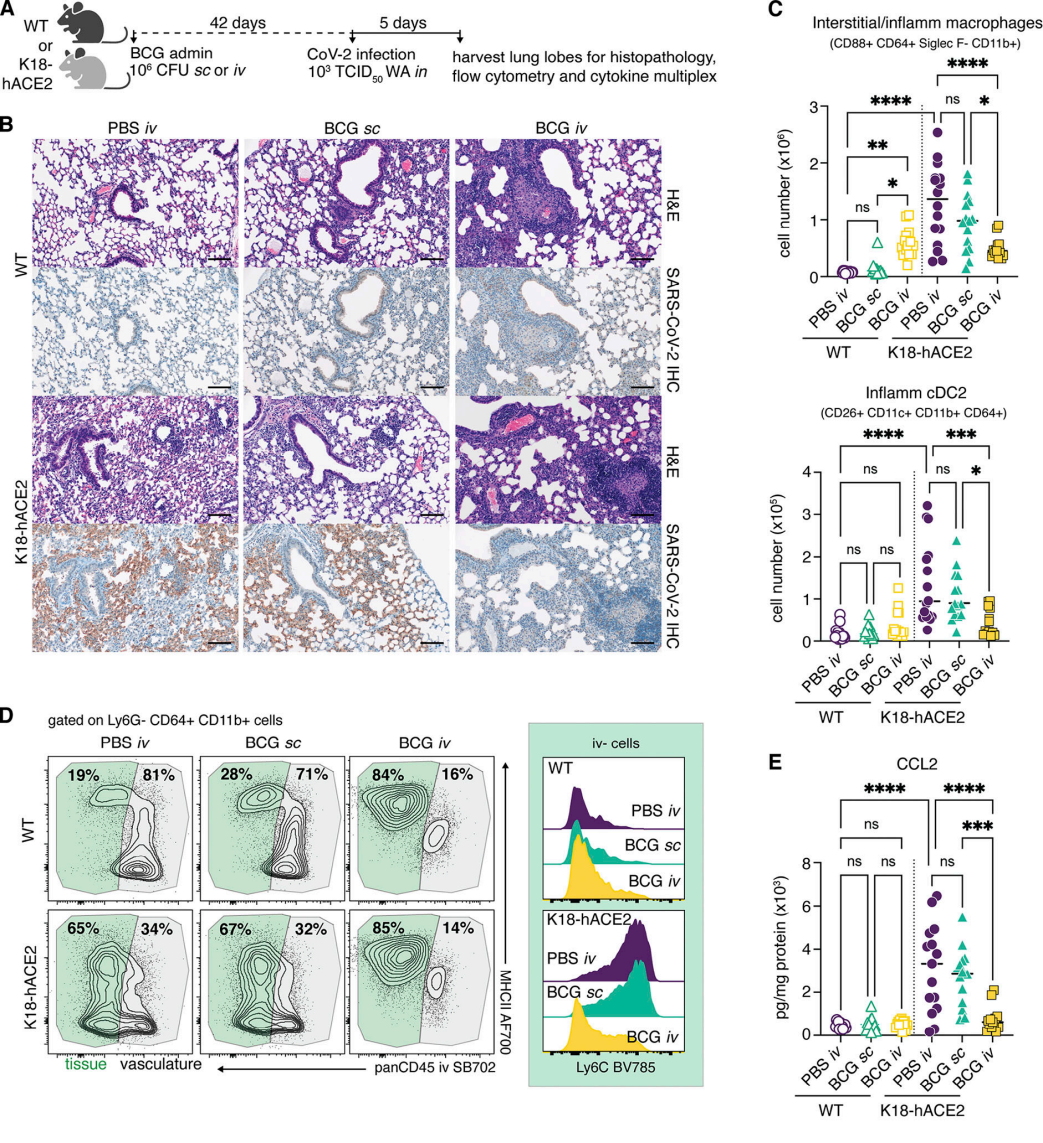
**SCV2-driven inflammatory cellular responses are dampened in mice previously inoculated with BCG i.v. (A–E)** K18-hACE2 mice or non-transgenic littermate controls (WT) were inoculated with 10^6^ CFUs BCG Pasteur by s.c. or i.v. injection. Control animals received the same volume of PBS i.v. At 42 d after BCG administration, mice were infected with 10^3^ TCID_50_ SCV2 (WA1/2020) by i.n. instillation. **(A)** Lungs were collected 5 d after viral challenge and assessed by histopathology, flow cytometry, and cytokine multiplexing. **(B)** Representative images of H&E-stained lung tissue sections (top panels) and sequential sections probed with an anti–CoV-2 nucleoprotein antibody (bottom panels). Scale bars: 100 μm. **(C)** Number of interstitial/inflammatory macrophages and inflammatory cDC2. **(D)** Contour plots depict MHCII expression by tissue-resident (i.v. stain negative; green) and vascular (i.v. stain positive; gray) CD64^+^ CD11b^+^ cells in the lung (left). Histograms show Ly6C expression by i.v.^−^ CD64^+^ CD11b^+^ cells (right). Data are concatenated from four to five mice per group and are representative of three independent experiments. **(E)** CCL2 levels in lung tissue homogenate standardized to total protein concentration. Statistical significance was assessed by one-way ANOVA with Tukey post hoc test. ns, P > 0.05; *, P < 0.05; **, P < 0.01; ***, P < 0.001; ****, P < 0.0001. Unless otherwise stated, data are pooled from three independent experiments each with four to five mice per group. Flow cytometry gating strategy is shown in Fig. S2. IHC, immunohistochemistry.

Lethal COVID-19 in humans is associated with the accumulation of proinflammatory myeloid cells within the lung tissue and airways (Delorey et al., 2021 *Preprint*; Melms et al., 2021). Similar associations have been made in murine models that can sustain a productive SCV2 infection, including the K18-hACE2 model (Winkler et al., 2020). To identify correlates of BCG i.v.–induced protection in SCV2-infected K18-hACE2 mice, we used histopathology, flow cytometric analysis, and cytokine multiplexing to assess the cellular composition and inflammatory environment of lung tissue from BCG i.v.–, BCG s.c.–, and PBS i.v.–treated animals 5 d after SCV2 challenge (Fig. 3 A). We also analyzed the lungs of non-transgenic littermate controls (referred to as WT hereafter) inoculated with BCG or PBS and challenged with SCV2 WA isolate. As WT animals cannot be productively infected with the WA strain of SCV2, these experimental groups allowed us to separate out BCG-induced responses from those driven by SCV2 infection.

**Figure S1.**
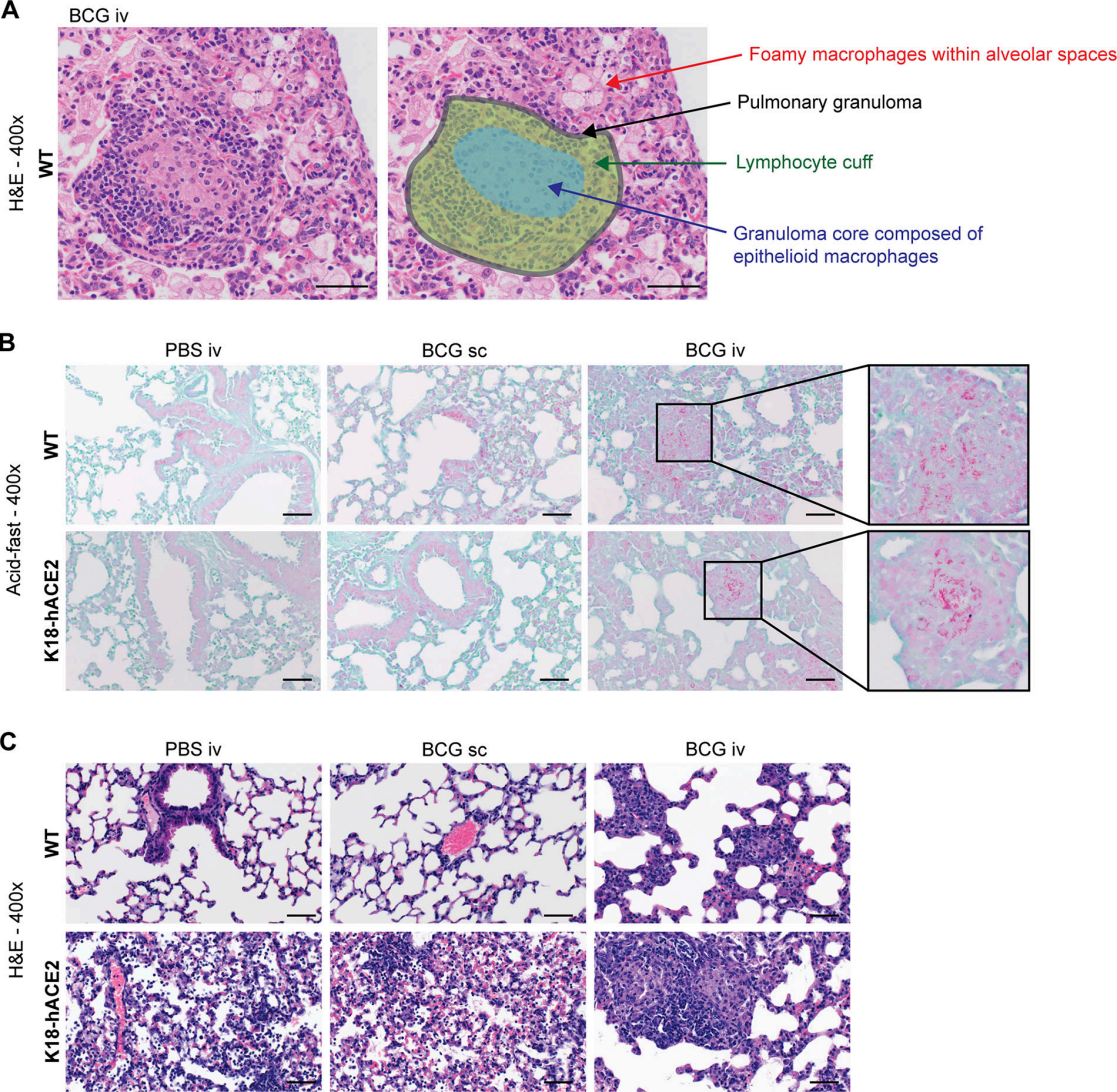
**Histological features of lungs from BCG i.v.–inoculated and SARS-CoV-2–infected animals. (A–C)** K18-hACE2 mice or non-transgenic littermate controls (WT) were inoculated with 10^6^ CFUs BCG Pasteur by s.c. or i.v. injection. Control animals received the same volume of PBS i.v. At 42 d after BCG administration, mice were infected with 10^3^ TCID_50_ SCV2 (WA1/2020) by i.n. instillation. Lungs were collected 5 d after viral challenge. **(A)** Example of a pulmonary granuloma and foamy macrophages observed in animals inoculated with BCG i.v. **(B)** Representative images of Ziehl–Neelsen–stained lung tissue sections. **(C)** Example images of H&E-stained lung tissue sections highlighting pneumonia observed in K18-hACE2 mice following SARS-CoV-2 infection. Scale bars: 50 μm.

Lungs from WT mice inoculated with BCG i.v. had moderate pulmonary pathology consisting of discrete, organized granuloma formation within alveolar septa, interstitial pneumonia, influx of foamy macrophages within alveolar spaces, and type II pneumocyte hyperplasia in 100% of mice examined (Fig. 3 B and Fig. S1 A). Granulomas were frequently perivascular in distribution, although some appeared to occlude terminal airways. As expected, acid-fast bacteria were located within macrophages making up the granuloma core. In line with the CFU data in Fig. 2 D, acid-fast bacteria were not found in the lung sections examined from mice inoculated with BCG s.c. (Fig. S1 B). Importantly, histopathology did not reveal any lesions consistent with coronaviral respiratory disease in WT (non-transgenic) animals (Fig. 3 B).

In contrast, lesions consistent with respiratory coronavirus infection were observed in 100% of non-BCG and BCG s.c.–inoculated K18-hACE2 mice infected with SCV2. Lungs from these animals displayed moderate interstitial pneumonia and leukocyte influx into adjacent alveolar spaces (Fig. S1 C). Bronchiolar disease was absent in all evaluated animals. Consistent with the histopathologic changes observed in the WT groups, interstitial pneumonia with influx of lymphocytes and macrophages was observed in both the BCG i.v.– and s.c.–vaccinated K18-hACE2 mice; however, the lesions associated with SCV2-driven pathology were largely absent from mice previously inoculated with BCG i.v. (Fig. 3 B and Fig. S1 C).

Immunohistochemistry for SCV2 nucleoprotein was performed to assess the distribution of viral antigen in association with lung pathology. Consistent with our RT-qPCR and TCID_50_ data, SCV2-specific immunoreactivity was prominent in sections from virus-challenged K18-hACE2 mice and was localized to numerous, widely dispersed type I and type II pneumocytes, as well as to macrophages within alveolar spaces. However, antigen distribution was limited in mice inoculated with BCG i.v., and was not observed adjacent to pulmonary granulomas (Fig. 3 B).

**Figure S2.**
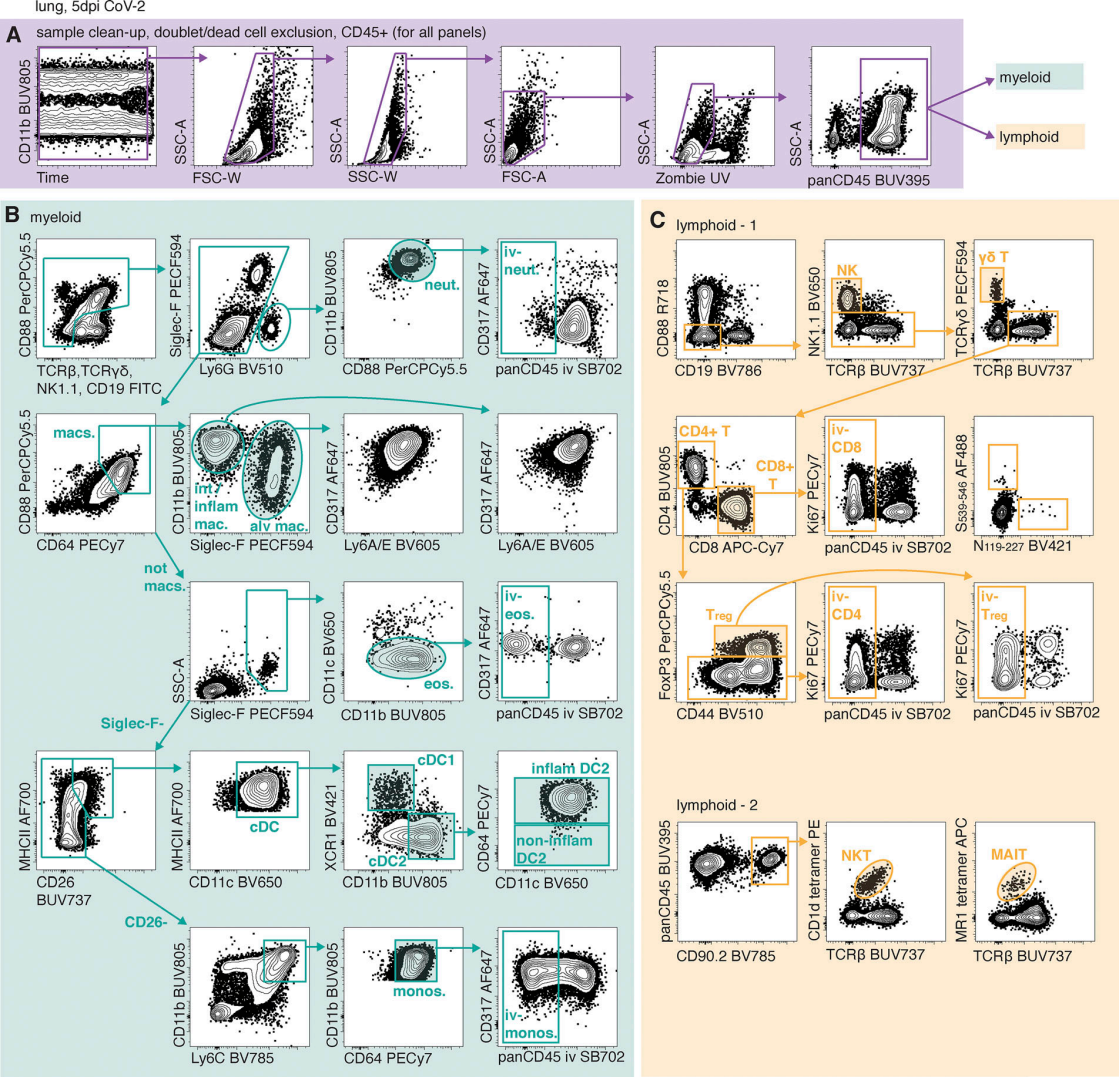
**Gating strategies for identifying cell types by flow cytometry. (A–C)** Single-cell suspensions were prepared from the lungs of animals i.v. injected with a fluorescently labeled panCD45 antibody 3 min before euthanasia to enable identification of cells located within the pulmonary vasculature. Cell suspensions were stained with cocktails of fluorescent antibodies and analyzed by flow cytometry. **(A)** Gating strategy to identify CD45^+^ live singlets. **(B)** Gating strategy to identify myeloid subsets by flow cytometry. **(C)** Gating strategies to identify lymphoid subsets by flow cytometry. alv, alveolar; eos, eosinophils; FSC-A, forward scatter area; FSC-W, forward scatter width; inflam, inflammatory; int, interstitial; mac, macrophage; monos, monocytes; neut, neutrophils; SSC-A, side scatter area; Treg, regulatory T cell.

**Figure S3.**
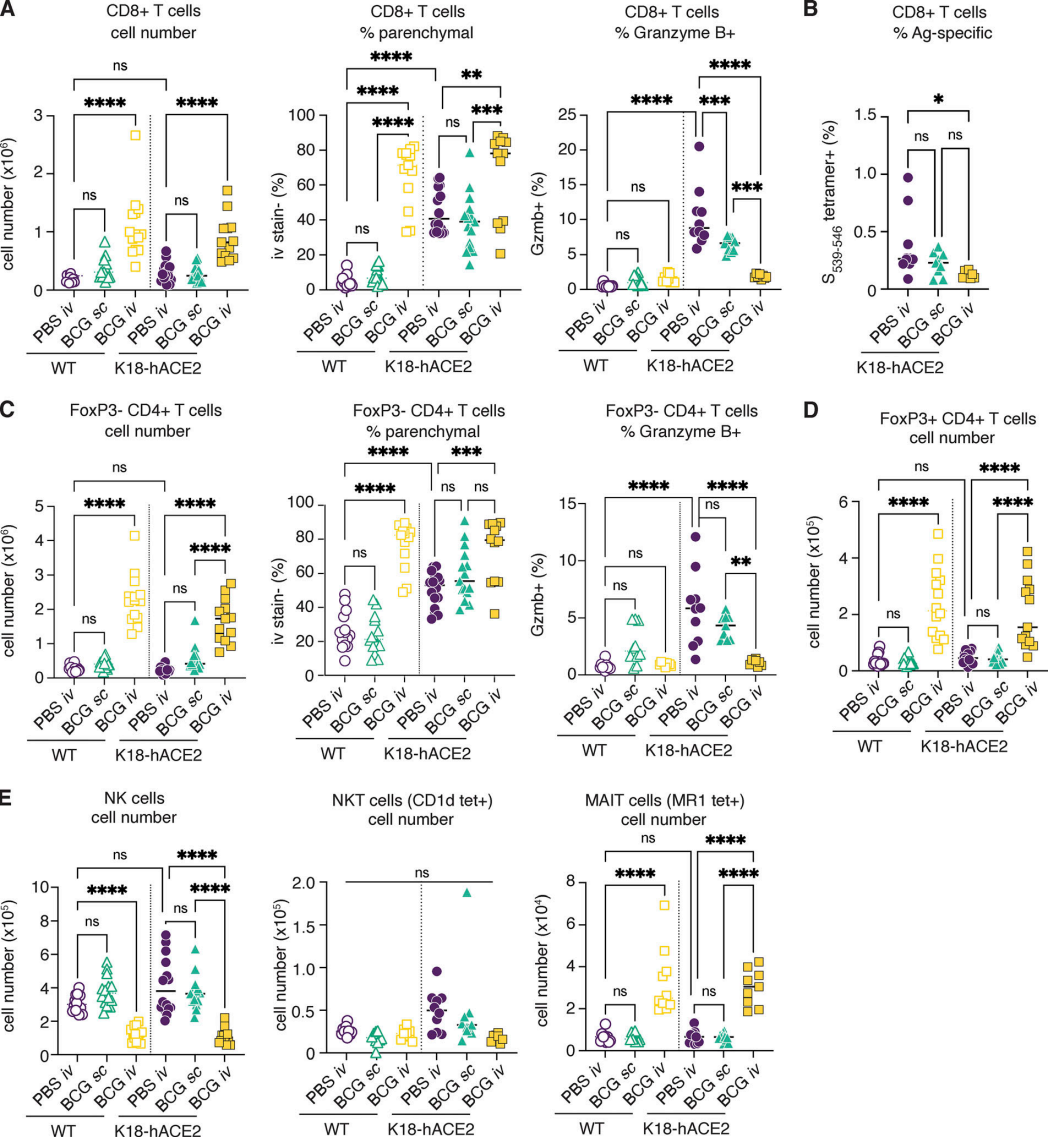
**Prior BCG i.v. administration limits bystander cytotoxic responses in SARS-CoV-2–challenged mice. (A–E)** K18-hACE2 mice or non-transgenic littermate controls (WT) were inoculated with 10^6^ CFUs BCG Pasteur by s.c. or i.v. routes. Control animals received the same volume of PBS i.v. At 42 d after BCG administration, mice were infected with 10^3^ TCID_50_ SARS-CoV-2 (WA1/2020) by i.n. instillation. Lungs were collected 5 d after viral challenge and assessed by flow cytometry. Gating strategy is shown in Fig. S2. **(A)** Number of CD8^+^ T cells, frequency of CD8^+^ T cells negative for i.v. stain, and frequency of CD8^+^ T cells expressing granzyme B. **(B)** Frequency of CD8^+^ T cells positive for the S(539-546) tetramer. **(C)** Number of FoxP3^−^ CD4^+^ T cells, frequency of FoxP3^−^ CD4^+^ T cells negative for i.v. stain, and frequency of FoxP3^−^ CD4^+^ T cells expressing granzyme B. **(D)** Number of FoxP3^+^ CD4^+^ T cells. **(E)** Number of NK, NKT, and MAIT cells. Statistical significance was assessed by one-way ANOVA with Tukey post hoc test. ns, P > 0.05; *, P < 0.05; **, P < 0.01; ***, P < 0.001; ****, P < 0.0001. Data are pooled from three independent experiments each with four to five mice per group. Ag, antigen; tet, tetramer.

Flow cytometric analysis of digested lung tissue at 5 dpi revealed significant upregulation of markers of activation and cell-cycle entry (Ki67, CD44, PD1, CD69) by conventional CD8^+^ and CD4^+^ T cells, natural killer T (NKT) cells, mucosal-associated invariant T (MAIT) cells, and γδ T cells in SCV2-infected K18-hACE2 mice injected with PBS i.v. or BCG s.c., suggesting that viral infection prompts a broad level of T cell activation (Fig. S2, Fig. S3, and Table S1). Intravenous injection of a fluorescently conjugated panCD45 antibody 3 min before euthanasia allowed us to distinguish cells located within the pulmonary vasculature (i.v. stain^+^) from cells within the lung parenchyma or airways (i.v. stain^−^; Anderson et al., 2014). This analysis revealed that SCV2 infection of PBS or BCG s.c.–treated K18-hACE2 mice promotes the recruitment of CD8^+^ T cells into the lung tissue, as evidenced by the striking increase in cells protected from i.v. staining, as well as a significant increase in granzyme B^+^ cells (Fig. S3 A). At this time point, SCV2 S (539–546)–specific CD8^+^ T cells were rare, suggesting that the majority of the observed CD8^+^ T cell response was nonspecific (Fig. S3 B). Similarly, SCV2-infected mice displayed an increase in parenchymal and granzyme B^+^ FoxP3^−^ CD4^+^ T cells (Fig. S3 C). Intravenous BCG administration alone was associated with a significant expansion of conventional CD8^+^, FoxP3^−^ CD4^+^, and FoxP3^+^ CD4^+^ T cells, as well as MAIT cells, which did not change after SCV2 infection (Fig. S3, A, C, D, and E). Interestingly, cytotoxic responses were not induced after SCV2 infection of mice that had previously received BCG i.v., as evidenced by low frequencies of granzyme B^+^ T cells and reduced numbers of NK cells compared with infected animals that received PBS i.v. or BCG s.c. (Fig. S3, A, C, and E).

Flow analysis of the myeloid compartment at 5 dpi showed significant expansion of CD88^+^ CD64^+^ CD11b^+^ Siglec F^−^ monocyte-derived macrophages in the lungs of SCV2-infected animals, consistent with previously published observations (Winkler et al., 2020). We also noted the appearance of a newly described population of inflammatory DC2s (CD88^−^ CD26^+^ CD11c^+^ MHCII^+^ XCR1^−^ CD11b^+^ CD64^+^) that are regulated by IFN and acquire functional characteristics of macrophages and cross-presenting DC1 (Fig. 3 C and Fig. S2; Bosteels et al., 2020). SCV2-induced Ly6G^−^ CD64^+^ CD11b^+^ cells, containing both DC2 and inflammatory/interstitial subsets, were largely protected from CD45 i.v. staining, indicating that they were indeed located within the tissue or airways (Fig. 3 D). Inoculation of WT animals with BCG i.v., but not s.c., also promoted the accumulation of myeloid cells within the lung, but the phenotype of these cells was that of resident interstitial Ly6C^lo^ MHCII^+^ macrophages rather than inflammatory Ly6C^+^ MHCII^+/−^ macrophages. The presence of this cell type within the lung tissue before SCV2 infection may contribute to the more effective viral control observed in BCG i.v.–inoculated mice given the reported protective role of CCR2^+^ monocytes/macrophages during SCV2 infection (Vanderheiden et al., 2021). However, it is important to note that productive SCV2 infection of animals previously inoculated with BCG i.v. did not promote further recruitment or expansion of inflammatory macrophages, monocytes, or DC2s, resulting in an overall lower number of inflammatory cells within the lungs compared with animals infected with SCV2 alone (Fig. 3 C). In this regard, analysis of lung homogenate revealed that SCV2 infection induced production of CCL2, a chemokine important for the recruitment of monocytes and DC2 precursors into the lung (Nakano et al., 2017; Serbina and Pamer, 2006). SCV2-induced CCL2 production was significantly lower in transgenic mice previously inoculated with BCG i.v. (Fig. 3 E). Together, these observations indicate that SCV2 infection results in altered proinflammatory and cytotoxic cellular responses in animals previously inoculated with BCG i.v. compared with control mice given either PBS i.v. or BCG s.c.

### SCV2-driven cytokine responses are dampened in BCG i.v.–inoculated mice

**Figure 4.**
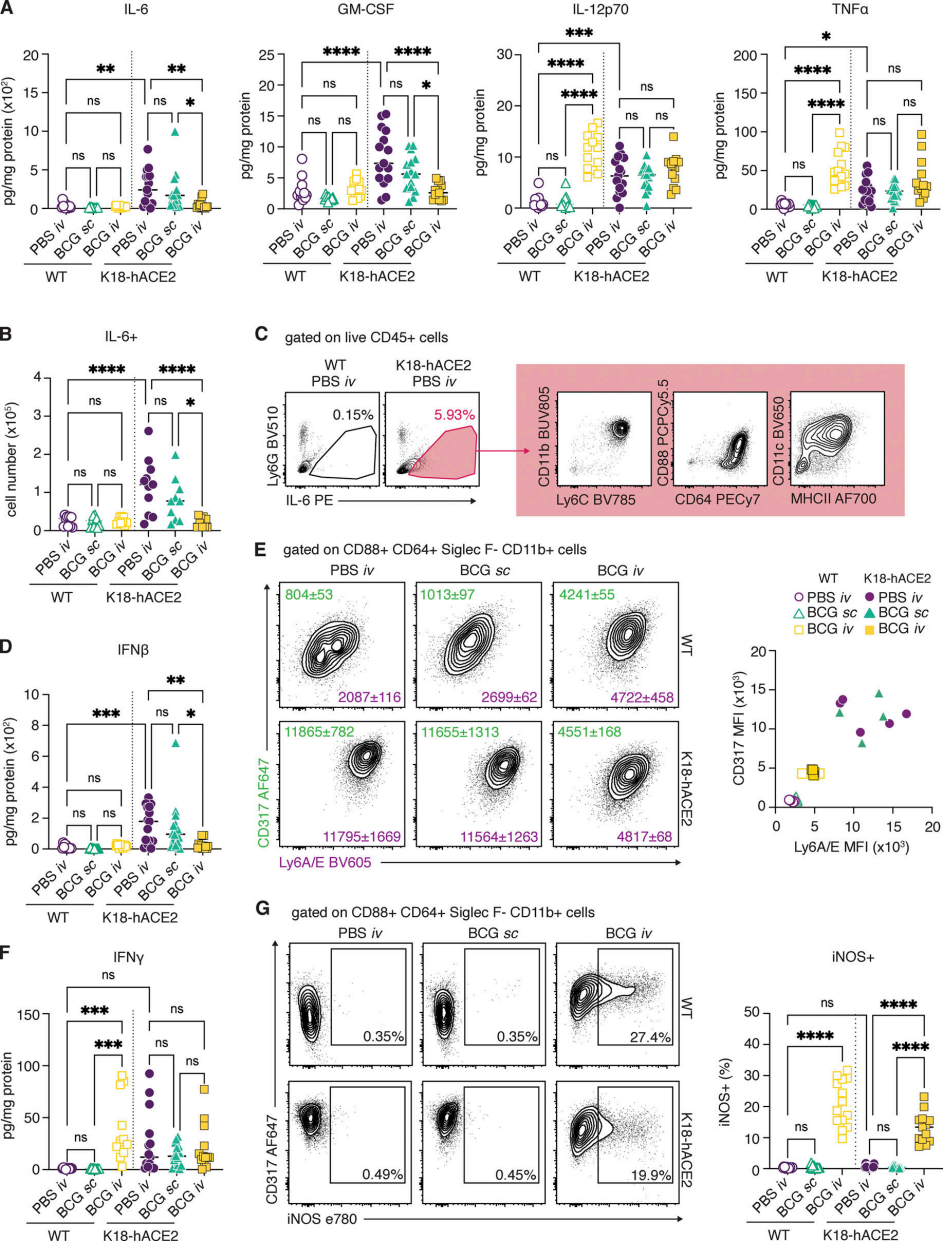
**BCG i.v. inoculation alters the inflammatory cytokine milieu of the lung following SCV2 challenge. (A–G)** K18-hACE2 mice or non-transgenic littermate controls (WT) were inoculated with 10^6^ CFUs BCG Pasteur by s.c. or i.v. routes. Control animals received the same volume of PBS i.v. At 42 d after BCG administration, mice were infected with 10^3^ TCID_50_ SCV2 (WA1/2020) by i.n. instillation. Lungs were collected 5 d after viral challenge and assessed by flow cytometry and cytokine multiplexing. **(A)** IL-6, GM-CSF, IL-12p70, and TNF-α levels in lung homogenate standardized to total protein concentration. **(B)** Number of IL-6^+^ cells as determined by flow cytometry. **(C)** Gating of IL-6^+^ cells from total CD45^+^ cells and surface marker expression of IL-6^+^ cells from K18-hACE2 PBS i.v. animals. Plots show data concatenated from five animals and are representative of two independent experiments. **(D)** IFN-β levels in lung homogenate standardized to total protein concentration. **(E)** Representative contour plots depict CD317 (green) and Ly6A/E (purple) expression by CD88^+^ CD64^+^ Siglec F^−^ CD11b^+^ macrophages (left). Median fluorescence intensity (MFI) ± SEM is indicated on the plots (*n* = 4–5). MFI values for individual animals are shown on the right. **(F)** IFN-γ levels in lung homogenate standardized to total protein concentration. **(G)** Representative contour plots depict CD317 and iNOS expression by CD88^+^ CD64^+^ Siglec F^−^ CD11b^+^ macrophages (left). Frequency of iNOS^+^ macrophages are shown on the right. Statistical significance was assessed by one-way ANOVA with Tukey post hoc test. ns, P > 0.05; *, P < 0.05; **, P < 0.01; ***, P < 0.001; ****, P < 0.0001. Unless otherwise stated, data are pooled from three independent experiments each with four to five mice per group. Flow cytometry gating strategy is shown in Fig. S2.

Because of the known association of severe COVID-19 with “cytokine storm” (Del Valle et al., 2020; Thwaites et al., 2021), we next assessed the effect of BCG i.v. inoculation on the cytokine milieu of lung tissue from SCV2-challenged animals. In keeping with published datasets, SCV2 infection drove a marked induction of proinflammatory cytokines, including IL-6, GM-CSF, IL-12p70, and TNF-α, in transgenic mice (Winkler et al., 2020), while BCG i.v. inoculation was associated with elevated IL-12p70 and TNF-α production before SCV2 infection (Wang et al., 1999). Conversely, IL-6 and GM-CSF levels were not induced by BCG i.v. alone and remained low following SCV2 challenge of these animals, resulting in significantly lower levels overall compared with PBS i.v. and BCG s.c. control animals infected with SCV2 (Fig. 4 A). Consistent with these observations, flow cytometric analysis showed fewer IL-6^+^ cells in SCV2-challenged mice previously inoculated with BCG i.v. compared with PBS i.v. or BCG s.c. control animals (Fig. 4 B). Of note, we identified CD88^+^ CD64^+^ CD11b^+^ CD11c^mid^ Ly6C^hi^ inflammatory macrophages to be the major source of IL-6 in SCV2-challenged mice (Fig. 4 C).

IFN-Is are important for control of viral infections but may also contribute to pathology in the context of SCV2 infection (Israelow et al., 2020). As expected, SCV2 challenge induced IFN-β production in the lungs of PBS i.v.– and BCG s.c.–treated animals, which was evident by direct measurement of IFN-β in lung homogenate (Fig. 4 D) and by the upregulation of IFN-inducible markers CD317 (BST2, PDCA1) and Ly6A/E (Sca1) on myeloid cells isolated from lung tissue (Fig. 4 E and Table S1). Importantly, BCG i.v. inoculation suppressed IFN-β induction, as well as CD317 and Ly6A/E upregulation, after SCV2 challenge of transgenic mice. The presence of an ongoing IFN response was suggested by the increased expression of CD317 and Ly6A/E on myeloid cells from WT mice inoculated with BCG i.v. compared with WT PBS i.v.– and BCG s.c.–treated animals. While IFN-β levels were low in all WT animals, a robust IFN-γ response was observed following BCG i.v. administration, suggesting that IFN-γ production contributes to the induction of IFN-stimulated responses in the lungs of these animals (Fig. 4 F). Indeed, a notable population of myeloid cells positive for the IFN-γ regulated enzyme inducible nitric oxide synthase (iNOS) was detected in the lungs of all BCG i.v. animals but was absent from the PBS i.v. and BCG s.c. groups (Fig. 4 G).

Together, the above findings demonstrate that prior BCG i.v. administration suppresses the SCV2-induced inflammatory cytokine response that is known to contribute to tissue damage in both humans and experimental animals infected with SCV2 (Del Valle et al., 2020; Israelow et al., 2020; Thwaites et al., 2021). This modulation of proinflammatory cytokines may be one of the mechanisms by which BCG i.v. protects transgenic animals from lethal challenge. Furthermore, the presence of an ongoing BCG-induced IFN-γ response in the lungs of i.v.-inoculated animals may support early control of SCV2 replication by innate cells, similar to the protective effects observed with IFN-I priming of airway epithelial organoids in vitro (Cheemarla et al., 2021).

### The suppression of SCV2-induced inflammatory response modules in BCG i.v.–inoculated mice is only partially related to diminished viral loads

**Figure 5.**
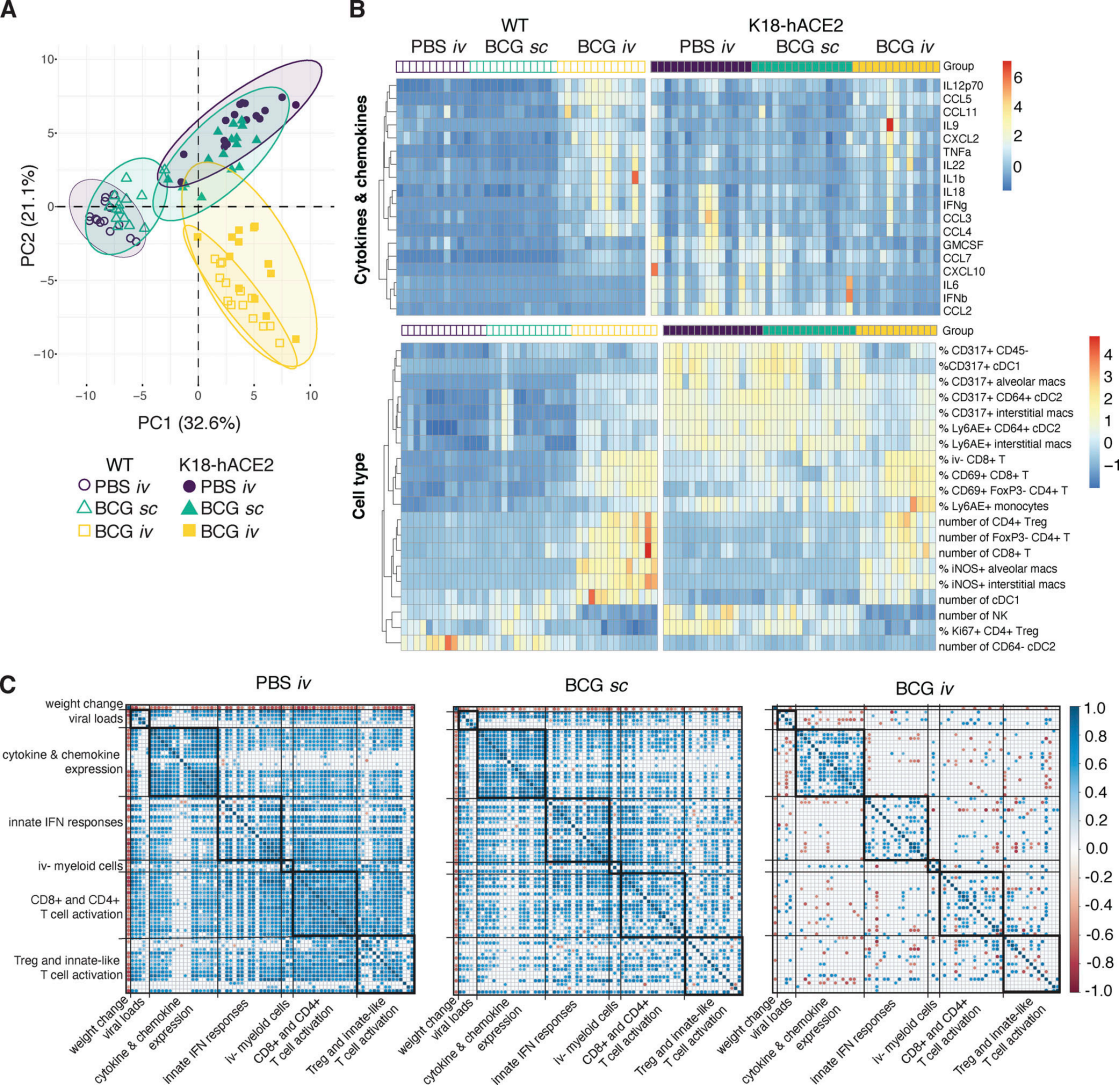
**Intravenous BCG inoculation suppresses inflammatory responses following SCV2 infection: lack of correlation with peak viral load. (A–C)** K18-hACE2 mice or non-transgenic littermate controls (WT) were inoculated with 10^6^ CFUs BCG Pasteur by s.c. or i.v. routes. Control animals received the same volume of PBS i.v. At 42 d after BCG administration, mice were infected with 10^3^ TCID_50_ SCV2 (WA1/2020) by i.n. instillation. Tissues were harvested for viral titers, flow cytometry analysis, and cytokine measurements 5 d after viral challenge. **(A)** PCA incorporating weight change; viral copies in nasal turbinates, lungs, and brain; cytokine levels in lung homogenate; and cell type frequencies and numbers in lung single-cell suspensions. The full dataset is shown in Table S1. **(B)** Hierarchical clustering of the top 10 cytokines and cell types that contribute to variation along the first and second components of the PCA is represented as a heat map. Experimental groups are indicated above the heat map. The full dataset is shown in Table S2. **(C)** Spearman’s correlation analyses of all parameters used in the PCA were performed for the PBS i.v., BCG s.c., and BCG i.v. groups separately. Positive and negative correlations are shown in blue and red as represented in the key, and only statistically significant correlations (adjusted P < 0.05) are indicated. macs, macrophages; Treg, regulatory T cell.

To more directly identify correlates of BCG i.v.–induced protection from SCV2-associated disease, we performed a multivariate analysis incorporating weight change, viral loads, flow cytometric parameters, and cytokine levels in all the individual animals studied at 5 dpi with SCV2. A principal component analysis (PCA) revealed overlapping signatures between PBS i.v. and BCG s.c. mice, whereas BCG i.v.–inoculated animals clustered separately (Fig. 5 A). SCV2-induced changes were evident for PBS i.v. and BCG s.c. animals along PC1, with activation of CD8^+^ T cells and their recruitment into the lung parenchyma as well as the upregulation of IFN-inducible markers by myeloid cells as key contributing factors (Fig. 5 B and Table S2). In contrast, BCG i.v. animals clustered together irrespective of SCV2 challenge. PC1 and PC2 separated the BCG i.v. group from the PBS i.v. and BCG s.c. animals, with PC2 representing variables uniquely driven by BCG i.v. inoculation (Fig. 5 A). The latter variables included increased numbers of CD4^+^ T cells and cDC1, reduced numbers of NK cells, and the induction of iNOS in myeloid cells (Fig. 5 B and Table S2). Hierarchal clustering of the cytokine and flow cytometry parameters that had strong contributions to variance along PC1 and PC2 highlighted the unique signatures present in animals that were i.v. BCG inoculated then challenged with SCV2, as well as the minimal changes observed between WT and transgenic animals within this treatment group (Fig. 5 B).

We next performed Spearman’s correlation analyses to assess the relationship among all parameters measured in our study. To do so, a pairwise comparison for each variable (e.g., viral titer, cytokine level, cell number) from the individual virus-challenged WT and K18-hACE2 mice was performed independently for the PBS i.v., BCG s.c., and BCG i.v. groups. Any significant correlations observed would therefore be a readout of an SCV2-induced response. Strong positive correlations between variables were observed in animals that received PBS i.v. The notable exception was weight change, which, as expected, was negatively associated with many of the variables measured. Viral loads positively correlated with induction of proinflammatory cytokines and chemokines, activation, and IFN priming of myeloid cells, as well as with cytotoxic activity by lymphocytes and their recruitment into the lung parenchyma (Fig. 5 C). Cytokine, chemokine, and cellular response modules that are coexpressed after SCV2 challenge were also identified from this analysis (Fig. 5 C and Table S2). For example, markers of CD4^+^ and CD8^+^ T cell activation, such as PD1, CD69, CD44, and Gzmb, displayed strong positive correlations. Similar modules and correlations were observed in animals that received BCG s.c., although these were less obvious than that seen with PBS i.v. controls, particularly for cellular responses. The majority of these significant correlations were absent from the cohort of animals that received BCG i.v. before challenge and, importantly, the associations with viral loads. This observation was particularly striking given that BCG i.v. had a minimal effect on peak viral loads at 3 dpi.

Collectively our findings suggest that while BCG i.v. reduces viral burden, this alone may not be sufficient to explain the pronounced inhibition of the SCV2 inflammatory response and protection from lethal challenge observed in K18-hACE2 animals. We therefore propose that a major effect of BCG i.v. exposure is to limit the pathological effects of the innate response to the virus and speculate that this outcome stems from the local effects of BCG-induced IFN-γ on the pulmonary epithelioid and myeloid compartments. The striking effects of BCG administration observed in our murine model experiments contrast with the negative or, at best, minor effects of BCG vaccination on COVID-19 susceptibility and severity in humans reported in published studies (Pépin et al., 2021; Tsilika et al., 2021* Preprint*). However, it is important to note that none of these clinical studies used BCG i.v. administration, which is currently not a clinically acceptable practice, although its use has been proposed in light of the strong protection observed against *M. tuberculosis* following BCG i.v. vaccination in nonhuman primates. In that *M. tuberculosis* model, protection is hypothesized to result from a strong antigen-specific T cell response within the lung parenchyma and airways (Darrah et al., 2020). Regardless of its lack of direct translational value, the experimental proof of concept that systemic delivery of BCG affords direct priming of pulmonary innate responses can trigger potent nonspecific protection against lethal SCV2 challenge may be of value in the discovery of previously unappreciated antiviral effector mechanisms and in the design of other strategies for COVID-19 prophylaxis that target the early response to this important pathogen.

## Materials and methods

### Mice

B6.Cg-Tg(K18-ACE2)2Prlmn/J hemizygous and noncarrier control animals (JAX34860) 7–9 wk of age were purchased from The Jackson Laboratory. C57BL/6 Thy1.1 mice 7–9 wk of age were acquired from the National Institute of Allergy and Infectious Diseases (NIAID) contract facility at Taconic Farms. Mice were housed under specific pathogen–free conditions with ad libitum access to food and water and were randomly assigned to experimental groups. All animal studies were performed in accordance with institutional guidelines and were conducted in Assessment and Accreditation of Laboratory Animal Care–accredited Biosafety Level 2 and 3 facilities at the NIAID/National Institutes of Health (NIH) using a protocol (LPD-99E) approved by the NIAID Animal Care and Use Committee. To make our findings consistent with earlier studies from our group on immune responses in BCG-vaccinated mice, female animals were used in all experiments. The responses of male animals were not tested.

### Virology

The SCV2 WA strain (Pango lineage A), originally isolated at the Centers for Disease Control and Prevention and representative of SCV2 viruses circulating early during the pandemic, was obtained from BEI Resources and propagated in tissue culture in Vero CCL81 cells (American Type Culture Collection). The SCV2 USA/CA_CDC_5574/2020 isolate, an α variant of concern (Pango lineage B.1.1.7), was obtained from BEI Resources and propagated in Vero cells overexpressing TMPRSS2, kindly provided by Dr. Jonathan Yewdell, (NIAID, Bethesda, MD). Vero cells were maintained in DMEM supplemented with GlutaMAX and 10% FBS. Vero-TMPRSS2 cells were maintained in DMEM supplemented with GlutaMAX, 10% FBS, and 250 μg/ml Hygromycin B Gold (InvivoGen). Virus stock production was performed under BSL-3 conditions by the NIAID SCV2 Virology Core using DMEM supplemented with GlutaMAX and 2% FBS. At 48 h after inoculation, culture supernatant and cells were collected and clarified by centrifugation for 10 min at 4°C. Supernatant was collected, aliquoted, and frozen at −80°C. Virus titers were determined by TCID_50_ assay in Vero E6 cells (CRL-1586; American Type Culture Collection) using the Reed–Muench calculation method. Full-genome sequencing was performed at the NIAID Genomic Core. The WA isolate stock used in this study was six passages from isolation and contained four single nucleotide polymorphisms compared with the reference sequence (MN985325.1): C23525T, C26261T, C26542T, and T28853A.

### BCG

BCG Pasteur from the Trudeau Collection was originally obtained from Dr. Sheldon Morris (Food and Drug Administration, Beltsville, MD) and maintained as laboratory stock by serial passage. BCG was propagated in 7H9 broth supplemented with oleic albumin dextrose catalase until mid-log phase. Bacteria were harvested, washed three times, and frozen down in aliquots until use. CFUs were enumerated by culturing on 7H11 agar for 3 wk at 37°C.

### Infections

BCG was prepared in PBS containing 0.05% Tween 80. A dose of 10^6^ CFUs/mouse was delivered by s.c. or i.v. injection. Control animals received the same volume of PBS with 0.05% Tween 80 by i.v. injection.

SCV2 infections were performed under BSL3 containment. Animals were anesthetized by isoflurane inhalation, and 10^3^ TCID_50_ SCV2 WA or 2.2 × 10^4^ TCID_50_ SCV2 B.1.1.7 was administered by i.n. instillation. After infection, mice were monitored daily for weight change and clinical signs of disease by a blinded observer who assigned each animal a disease score based on the following criteria: 0, no observable signs of disease; 1, hunched posture, ruffled fur, and/or pale mucous membranes; 2, hunched posture and ruffled fur with lethargy but responsive to stimulation, rapid/shallow breathing, and dehydration; and 3, moribund.

### Determination of viral copies and ACE2 expression by qPCR

Lung, brain, and nasal turbinates were homogenized in TRIzol, and RNA was extracted using the Direct-zol RNA Miniprep kit following the manufacturer’s instructions. E gene gRNA was detected using the QuantiNova Probe RT-PCR Kit and protocol and primers (forward primer: 5′-ACA​GGT​ACG​TTA​ATA​GTT​AAT​AGC​GT-3′; reverse primer: 5′-ATA​TTG​CAG​CAG​TAC​GCA​CAC​A-3′) and probe (5′-FAM-ACACTAGCCATCCTTACTGCGCTTCG-3IABkFQ-3′) as previously described (Corman et al., 2020). The standard curve for each PCR run was generated using the inactivated SARS-CoV2 RNA obtained from BEI Resources (NR-52347) to calculate the viral copy number in the samples. Human ACE2 transgene expression in the lung was quantified using the TaqMan probe (assay ID: Hs01085333_m1 FAM) and Fast Advanced Master Mix following the manufacturer’s instructions. The expression of mouse GAPDH gene (assay ID: Mm99999915_g1 VIC) was used as internal control, and the relative expression of hACE2 was calculated using the 2^−ΔΔCT^ method. Identical lung and brain portions were used for all experiments to generate comparable results.

### Determination of viral titers by TCID_50_ assay

Viral titers from lung and brain homogenate were determined by plating in triplicate on Vero E6 cells using 10-fold serial dilutions. Plates were stained with crystal violet after 96 h to assess cytopathic effect. Viral titers were determined using the Reed–Muench method.

### Preparation of single-cell suspensions from lungs

Lung lobes were diced into small pieces and incubated in RPMI containing 0.33 mg/ml Liberase TL and 0.1 mg/ml DNase I (both from Sigma-Aldrich) at 37°C for 45 min under agitation (200 rpm). Enzymatic activity was stopped by adding FCS. Digested lung was filtered through a 70-μm cell strainer and washed with RPMI. Red blood cells were lysed with the addition of ammonium-chloride-potassium buffer (Gibco) for 5 min at room temperature. Cells were then washed with RPMI supplemented with 10% FCS. Live cell numbers were enumerated using ViaStain acridine orange propidium iodide staining on a Cellometer Auto 2000 Cell Counter (Nexcelom). For assessment of cytokine production, single-cell suspensions were incubated in FCS-supplemented RPMI in the presence of 1× Protein Transport Inhibitor Cocktail (eBioscience) for 5 h.

### Flow cytometry

To label cells within the pulmonary vasculature for flow cytometric analysis, 2 μg anti-CD45 (30-F11; Invitrogen) was administered i.v. 3 min before euthanasia. Single-cell suspensions prepared from lungs were washed twice with PBS before incubating with Zombie UV Fixable Viability Dye and TruStain FcX (clone 93; both from BioLegend) for 15 min at room temperature. Cocktails of fluorescently conjugated antibodies diluted in PBS containing 2 mM EDTA, 0.01% sodium azide, 2% FCS, and 10% Brilliant Stain Buffer (BD Biosciences) were then added directly to cells and incubated for a further 20 min at room temperature. Anti-CD4 (GK1.5), anti-CD11b (M1/70), and anti-CD26 (H194-112) were from BD OptiBuild. Anti-CD19 (GL3), anti-CD45 (30-F11), anti–Siglec F (E50-2440), anti–TCR-β chain (H57-597), and anti–TCR-γδ (GL3) were from BD Horizon. Anti–CD8-α (53-6.7) was from Invitrogen. Anti-CD11c (N418), anti-CD19 (6D5), anti-CD44 (IM7), anti-CD64 (X54-5/7.1), anti-CD69 (H1.2F3), anti-CD86 (GL-1), anti-CD88 (20/70), anti-CD279 (PD1, clone 29F.1A12), anti-CD317 (BST2, clone 927), anti-IA/IE (MHCII, clone M5/114), anti-NK1.1 (PK136), anti-Ly6A/E (Sca-1, clone D7), anti-Ly6C (HK1.4), anti-Ly6G (1A8), anti–TCR-β chain (H57-597), anti–TCR-γδ (GL3), and anti-XCR1 (ZET) were from BioLegend. SCV2 S(539–546) and N(119–227) tetramers were from the NIH Tetramer Core.

Cells were incubated in eBioscience Transcription Factor Fixation and Permeabilization solution (Invitrogen) for 2–18 h at 4°C and stained with cocktails of fluorescently labeled antibodies against intracellular antigens diluted in eBioscience Permeabilization Buffer (Invitrogen) for 20 min at room temperature followed by 20 min at 4°C. Anti-granzyme B (GB11) and anti-Ki67 (B56) were from BD Pharmingen. Anti-FoxP3 (FJK-16s), anti-NOS2 (CXNFT), and anti-Tbet (4B10) were from Invitrogen. Anti–IL-6 (MP5-20F3) and anti–TNF-α (MP6-XT22) were from BioLegend.

Compensation was set in each experiment using UltraComp eBeads (Invitrogen), and dead cells and doublets were excluded from analysis. All samples were collected on a FACSymphony A5 SORP flow cytometer (BD Biosciences) and analyzed using FlowJo version 10 software (BD Biosciences). A minimum of 1,000 events were analyzed per gated population.

### Multiplex cytokine array

Cytokines were assessed in lung homogenate using a ProcartaPlex Luminex kit (Thermo Fisher Scientific) according to the manufacturer’s instructions and measured using a MAGPIX Instrument (R&D Systems). Total protein was measured by Pierce Protein Assay (Thermo Fisher Scientific). Cytokine levels were standardized to total protein content.

### Histology

Tissues were fixed in 10% neutral buffered formalin for 48–72 h and transferred into 30% sucrose and embedded in paraffin. Embedded tissues were sectioned at 5 μm and dried overnight at 42°C before staining. Specific anti-CoV immunoreactivity was detected using an SCV2 nucleoprotein antibody (GenScript) at a 1:1,000 dilution. The secondary antibody was the ImmPRESS-VR Anti-Rabbit IgG Polymer (cat. no. MP-6401; Vector Laboratories). The tissues were then processed for immunohistochemistry using the DISCOVERY ULTRA automated stainer (Ventana Medical Systems) with a ChromoMap DAB Kit (cat. no. 760-159; Roche Tissue Diagnostics). All tissue slides were evaluated by a board-certified veterinary pathologist.

### Statistical analyses

In all cases, statistical analyses were performed on pooled data from two to three independent experiments, each with four to five mice per group. All data points are shown on the graphs, and no animals were excluded. Data for each of the individual experiments within the pool are presented in Table S1.

P values were determined by Student’s unpaired *t* test or Mann–Whitney test when comparing two groups or by one-way ANOVA with Tukey’s post hoc test or Kruskal–Wallis test with Dunn’s post hoc test when comparing three or more groups using GraphPad Prism 9 software. P < 0.05 was considered statistically significant.

Weight change, viral copies, and multiplex cytokine and flow cytometry measurements generated from WT and K18-hACE2 mice for the three experimental groups (PBS i.v., BCG s.c., and BCG i.v.) from three independent experiments were compiled and PCA performed and visualized in R version 4.1.0 with packages FactoMineR and factoextra. The top 10 variables contributing to the first two principal components were identified for the cytokine and flow cytometry data, and these measurements were normalized, clustered, and visualized as a heat map using the R package pheatmap. Pairwise Spearman’s correlation values were calculated for the same readouts as used for PCA for each of the three experimental groups. Correlations that were found to be significant following Benjamini–Hochberg correction for multiple testing were visualized using the R packages RcmdrMisc and corrplot.

### Figure visualization

Figures were generated in Adobe Illustrator and R, incorporating images from BioRender.com.

### Online supplemental material

Fig. S1 shows examples of histopathological changes observed in the lungs of BCG-injected and/or SARS-CoV-2–challenged K18-hACE2 mice, as well as acid-fast staining for mycobacteria in pulmonary tissue sections. Fig. S2 shows the gating strategy employed to identify various immune cell types by flow cytometry. Fig. S3 shows quantification of lymphoid cell populations from the lungs of WT and K18-hACE2 animals inoculated with PBS i.v., BCG s.c., or BCG i.v. and then challenged with SARS-CoV-2; cytotoxic responses (number of NK cells and granzyme B^+^ T cells) were reduced in mice that received BCG i.v. before challenge with SARS-CoV-2 compared with control animals. Table S1 contains all the raw data used for the multivariate analysis, separated by experiment. Table S2 summarizes the statistically significant drivers of variation along PC1 and PC2 for the PCA shown in Fig. 5 A.

## Supplementary Material

Table S1 contains all the raw data used for multivariate analysis, separated by experiment.

Table S2 summarizes the statistically significant drivers of variation along PC1 and PC2 for the PCA shown in Fig. 5 A.
